# Endothelial Dysfunction in Diabetes Mellitus: Possible Involvement of Endoplasmic Reticulum Stress?

**DOI:** 10.1155/2012/481840

**Published:** 2012-02-28

**Authors:** Basma Basha, Samson Mathews Samuel, Chris R. Triggle, Hong Ding

**Affiliations:** Department of Pharmacology, Weill Cornell Medical College in Qatar, Education City, Qatar Foundation, P.O. Box 24144, Doha, Qatar

## Abstract

The vascular complications of diabetes mellitus impose a huge burden on the management of this disease. The higher incidence of cardiovascular complications and the unfavorable prognosis among diabetic individuals who develop such complications have been correlated to the hyperglycemia-induced oxidative stress and associated endothelial dysfunction. Although antioxidants may be considered as effective therapeutic agents to relieve oxidative stress and protect the endothelium, recent clinical trials involving these agents have shown limited therapeutic efficacy in this regard. In the recent past experimental evidence suggest that endoplasmic reticulum (ER) stress in the endothelial cells might be an important contributor to diabetes-related vascular complications. The current paper contemplates the possibility of the involvement of ER stress in endothelial dysfunction and diabetes-associated vascular complications.

## 1. Introduction

Diabetes mellitus (DM) is a growing metabolic disease that continues to be a leading health problem worldwide. The World Health Organization (WHO) has estimated that there are currently 346 million people affected by diabetes worldwide and anticipates that diabetes-related deaths would double by 2030 [1]. These figures highlight the importance of continued research and the need for novel methods to both prevent and treat this pandemic.

The adverse long-term effects of DM involve many organ systems and are associated with a complex pathology involving a large number of secondary cellular and subcellular changes. While diabetes management has largely focused on control of hyperglycemia, the rising burden of this disease is mainly correlated to its vascular complications [2, 3]. This is reflected by a 4-fold increase in the incidence of coronary artery disease, a 10-fold increase in peripheral vascular disease, and a 3- to 4-fold higher mortality rate with as much as 75% of diabetics ultimately dying from vascular disease [4]. Type II diabetes differs principally from type I diabetes in that it is accompanied by a period of hyperinsulinemia and is characterized by late as opposed to early onset of hyperglycemia. In type I DM, vascular involvement (through endothelial dysfunction) occurs as a result of metabolic insult/hyperglycemia, while in type II DM, endothelial dysfunction plays a more direct role, and is aggravated by, rather than caused by, hyperglycemia [5].

The high glucose-induced “*oxidative stress*” and “*endoplasmic reticulum (ER) stress*” (which might be dependent on oxidative stress) of the endothelium may play major roles in the initiation and progression of cardiovascular clinical manifestations in diabetes. The following sections of this paper will provide evidence on the molecular mechanisms of endothelial dysfunction in diabetes with special reference to the role of hyperglycemia/oxidative stress-induced ER stress in the endothelium.

## 2. Endothelial Dysfunction and Vascular Complications in Diabetes

Endothelial cells line the internal lumen of all the vasculature and serve as an interface between circulating blood and vascular smooth muscle cells (VSMCs) [6]. Apart from being the key participant during the process of angiogenesis, these dynamic structures can actively regulate basal vascular tone and vascular reactivity in physiological and pathological conditions. They respond to mechanical forces and neurohumoral mediators by releasing a variety of relaxing and contracting factors such as nitric oxide (NO) and prostacyclin [7]. The balance between the vasodilatation and vasoconstriction is maintained by the endothelium, and the disruption of this balance leads to endothelial dysfunction and causes damage to the arterial wall [8, 9]. Endothelial cells are also responsible for the maintenance of blood fluidity and restoration of vessel wall integrity (when injured) to avoid bleeding [7]. Endothelial cell-derived factors also are critical mediators of VSMC growth and inflammation [10].

Loss of function/regulation of function of the endothelium (endothelial dysfunction) in a basal state or after activation may be a critical and initiating factor in the development of diabetic micro- and macrovascular disease [2, 11]. Blood glucose levels continue to be the principal link between diabetes and vascular disease. In diabetics and normal subjects, results of glucose tolerance tests have shown rapid loss of brachial artery endothelium-dependent vasodilatation—with fast recovery in normal subjects and a slower recovery in diabetics [12]. This emphasizes that even in nondiabetics, postprandial exposure to elevated blood glucose can disturb the endothelium-dependent regulation of blood flow [13].

The microvascular complications in diabetes include retinopathy, nephropathy, and neuropathy, while macrovascular complications are manifested in ischemic heart disease, stroke, and peripheral vascular disease [3, 14]. “*Aberrant*” angiogenesis (one of the main functions of endothelial cells) is one of the main reasons for these vascular complications associated with diabetes [15]. However, it must be noted that diabetic vascular complications associated with aberrant/altered angiogenesis may either be caused by excessive angiogenesis (retinopathy, nephropathy) or deficient angiogenesis (impaired wound healing, impaired collateral vessel formation, neuropathy, embryonic vasculopathy, and transplant rejection) [15].

Several diabetes-related studies have identified an array of molecular entities and pathways that influence endothelial function and reported how altered endothelial function and changes in endothelial cell-derived factors can lead to vascular complications in a hyperglycemic setting. There is, however, no distinct pathway that causes vascular disease in diabetes, due to the simple fact that the dysregulation of glucose homeostasis (the defining feature of diabetes) can in itself occur either in the absence or presence of insulin, not to mention other frequent comorbid diseases such as hypertension and obesity. Therefore, the cellular processes underlying endothelial dysfunction are not yet clearly understood, and multiple mechanisms are probably involved. A better understanding of endothelial dysfunction in a diabetic setting would aid in the search for novel approaches in the prevention and treatment of diabetes vascular disease.

## 3. Etiology of Endothelial Dysfunction in Diabetes

Type II diabetes is characterized by three main metabolic disturbances (triggers): (1) *hyperlipidemia*, (2) early *hyperinsulinemia,* and (3) hyperinsulinemia followed by pancreatic *β*-cell failure leading to *hyperglycemia* [16]. Each of these metabolic disturbances acts as “triggers” eventually causing endothelial dysfunction through the influence of different “mediator” molecules [17–19]. In a clinical setting it might be difficult to validate how much damage is caused in terms of endothelial dysfunction by each of these metabolic changes. However, several lines of evidence point to the fact that “oxidative stress” caused by these metabolic changes plays a key role in endothelial dysfunction [7, 20]. 

**Figure 1:**
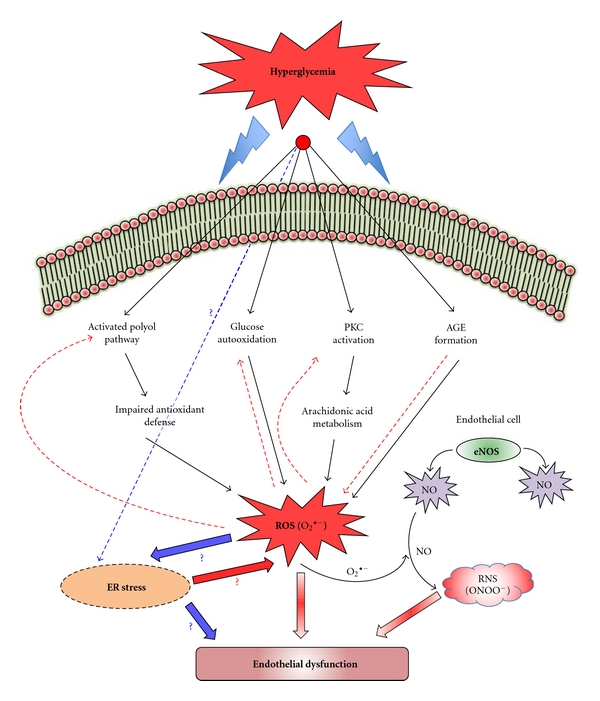
*Hyperglycemia-induced oxidative stress and endothelial dysfunction (possible role of ER stress)*. High glucose levels in circulation can divert glucose into alternative biochemical pathways leading to the increase in advanced glycation end products (AGEs), glucose autooxidation, hexosamine and polyol flux, and activation of classical isoforms of protein kinase C, that are considered to be the mediators of hyperglycemia-induced cellular injury. Many different pathways involved in hyperglycemia-mediated endothelial dysfunction induced by hyperglycemia lead to considerable generation of reactive oxygen species (ROS), which is responsible for the oxidative stress. The excessive ROS so formed can then aggravate cellular injury by promoting activation of the biochemical pathways (red dotted arrows) that initiate ROS generation in the first place as a response to hyperglycemia, thus completing a vicious cycle. Superoxide anion (O_2_^∙−^) can also react with NO to yield peroxynitrite which is also known to be a mediator of endothelial dysfunction. It is still unclear whether the ER stress response can be initiated as a direct response to the increasing load on protein synthesis and maturation due to hyperglycemia or due to the hyperglycemia-associated oxidative stress. The possibility that ER stress response also can lead to excessive ROS formation cannot be ruled out.

Among these metabolic changes hyperglycemia has been recognized as the primary cause in the pathogenesis of diabetic vascular disease and other complications. Hyperglycemia-induced increase in glucose oxidation and mitochondrial generation of superoxide anion (O_2_^∙−^) in turn leads to DNA damage and activation of poly (ADP ribose) polymerase (PARP) as a reparative enzyme [16, 21]. PARP-induced ADP ribosylation of glyceraldehyde phosphate dehydrogenase (GAPDH) then diverts glucose from its glycolytic path into alternative biochemical pathways leading to increase in advanced glycation end products (AGEs), hexosamine and polyol flux, and activation of classical isoforms of protein kinase C, that are considered the mediators of hyperglycemia-induced cellular injury [16, 21–25]. Though there are several different pathways involved in hyperglycemia-mediated endothelial dysfunction, evidence suggests that all these different pathways/mechanisms that are induced by hyperglycemia lead to considerable generation of reactive oxygen species (ROS), which is responsible for the oxidative stress; however, metabolism of glucose may not be a requirement for the generation of ROS [26]. Oxidative stress or the increase in the levels of ROS in the biological system is known to occur due to increased activity of the ROS-generating systems and/or decreased antioxidant defense mechanisms [27]. The excessive ROS so formed can then aggravate cellular injury by promoting activation of the biochemical pathways (autooxidation of glucose, AGE formation, activation of the polyol pathway, and stimulation of the eicosanoid metabolism) that initiate ROS generation in the first place as a response to hyperglycemia, thus completing a vicious cycle [28] (Figure 1).

The excessive ROS generation is known to impair endothelial nitric oxide synthase (eNOS) activity and NO production thereby affecting endothelium-dependent vasodilation [23]. Hyperglycemia-induced oxidative stress has also been associated with increased endothelial cell apoptosis *in vitro* and *in vivo *[29]. Several antioxidant therapies were found to improve or normalize the endothelium-dependent responses in different models of diabetes and hyperglycemia as well as significantly decrease the hyperglycemia-induced apoptosis [28–30]. Both experimental and clinical evidence thus suggest compromised endothelial function in diabetic conditions and identified hyperglycemia and/or hyperglycemia-mediated oxidative stress as the major causative factor for the cardiovascular pathological conditions that follow [7, 27, 28]. 

## 4. Molecular Basis of Endothelial Dysfunction in Diabetes—Current Understanding

Endothelial cells have multiple physiological functions, and therefore alterations in endothelial cell function may affect one or more of these systems, either simultaneously or at distinct time periods [7]. Thus, either an increase or a decrease in any of the endothelial cell-related chemical messengers and/or alterations in any of the endothelial cell-related functions may contribute to the development of endothelial dysfunction [7].

In type I diabetic patients, reports of poor vasodilatation, increased blood levels of von Willebrand factor (vWF), thrombomodulin, selectins, type IV collagen, and so forth are indicators of endothelial cell dysfunction [7]. These pathological changes can then induce alterations in the vasculature and is known to support the progression of the disease condition. endothelin-1 (ET-1), a potent vasoconstrictor and mitogen produced by endothelial cells that act on VSMCs, is significantly increased in hyperglycemic/diabetic conditions and plays a critical role in the development of vascular diseases [31, 32]. Additionally, increased activation of protein kinase C (PKC), angiotensin II (ANG-II), increased levels of advanced glycation end products (AGEs), increased plasminogen activity inhibitor-1 (PAI-1), and so forth have been related to endothelial dysfunction in diabetes.

Increased inflammatory activation has also been linked to diabetic vascular complications [5]. Human aortic endothelial cells (HAECs) exposed to transient hyperglycemia have exhibited epigenetic changes in the p65 subunit of NF*κ*B gene, with increased expression of monocyte chemoattractant protein -1 (MCP-1), vascular cell adhesion molecule-1 (VCAM-1), intercellular adhesion molecule-1 (ICAM-1), interleukin-6 (IL-6), and inducible nitric oxide synthase (iNOS) [33, 34]. Also, a rise in the soluble forms of VCAM-1 and ICAM-1 (leukocyte adhesion molecules) has been observed in diabetic patients and is associated with an increased cardiovascular disease risk [35, 36]. Furthermore, there is a moderate elevation in plasma C-reactive protein levels in diabetic and atherosclerotic patients, which could be a manifestation of low-grade chronic inflammation of vessels [37–40].

*In vitro* and *in vivo* studies demonstrated that the activity of eNOS and generation of nitric oxide (NO) are significantly reduced in endothelial cells exposed to a hyperglycemic environment [7, 9, 23, 41]. It has been demonstrated that mouse microvascular endothelial cells (MMECs) develop oxidative stress after exposure to high glucose [42]. NADPH oxidase is activated by high glucose and acts as the primary source of ROS (specifically O_2_^∙−^), and this activation promotes eNOS uncoupling and higher production of H_2_O_2_ and superoxide. The excess O_2_^∙−^ may also quench NO thereby reducing its bioavailability leading to reduced endothelium-dependent vasodilatation [43–45]. Reports have also suggested that hyperglycemia-induced O_2_^∙−^ production is accompanied by an increase in NO formation as a result of the activation of inducible NOS (iNOS) [21]. The O_2_^∙−^ and NO react to yield peroxynitrite (ONOO^−^), a strong oxidant, which in turn is known to contribute significantly to endothelial dysfunction [46–48] (Figure 1). Peroxynitrite oxidizes tetrahydrobiopterin (BH_4_), an essential cofactor in the regulation of eNOS and iNOS levels in endothelial cells, and its levels are affected by acute rises in plasma glucose [21, 42, 49–51]. BH_4_ is known to stabilize the dimeric forms of NOS, which would otherwise remain as a monomer [21, 52–54]. BH_4_ is therefore of prime importance in the maintenance of endothelial function, and it has been shown to be affected early in both humans and mice in type II diabetes [44, 55].

Recent evidence suggests that hyperglycemia-induced endothelial cell dysfunction may occur secondary to increased oxidant stress and a concomitant increase in ER stress [56, 57]. Although oxidative stress can induce ER stress, it is still unclear whether these cellular responses are causally linked or two independent effects of high glucose exposure. The following sections of this paper dwell upon the possible role of ER stress in endothelial dysfunction in a diabetic milieu.

## 5. ER Stress: General Overview

Apart from playing a key role as a central eukaryotic membranous organelle, formed in continuity with the outer membrane of the nuclear envelope and responsible for synthesis, folding, and maturation of membrane and secreted proteins, lipid biosynthesis, and calcium storage, the endoplasmic reticulum (ER) acts as the prime quality control centre and signal transducing entity that can sense and respond to changes in cellular homeostasis [58, 59]. The intra-ER milieu provides a Ca^2+^- rich, unique oxidizing environment, which is critical for the formation of disulphide bonds and for protein folding and assembly into its precise native conformations prior to transport to the Golgi compartment [58, 59]. In addition, accuracy in protein folding is also monitored by the many Ca^2+^-dependent molecular chaperones, which stabilize the protein folding intermediates [60].

The entry of unfolded polypeptide chains, as they are synthesized, into the ER remains largely variable, changing rapidly in response to the different ongoing cellular process, the environment, and the physiologic state of the cell [61, 62]. As such the ER must be highly receptive to these changes and critically respond in the best interest of maintaining cell integrity and normal function. The normal physiological state of the ER is challenged when the influx of the nascent unfolded polypeptides exceeds the processing capacity of the ER (ER stress) [63]. In such a scenario the cell responds to the increasing presence of unfolded proteins within the ER through the activation of an intricate set of integrated signaling pathways that relay information from the ER to the cytosol and the nucleus, aiming to restore normal ER and cellular functions [63–65]. In fact, a cell's life and death decisions are made by these pathways depending on whether the ER stress is resolved or not. These pathways, known as the *unfolded protein response (UPR)* or *ER stress response* are thus critical for normal cellular homeostasis, development of the organism, and are also known to play major roles in the pathogenesis of many diseases such as diabetes, obesity, inflammation, cardiovascular disorders, viral infections, neurodegeneration, and cancer [64, 66–71]. 

**Figure 2:**
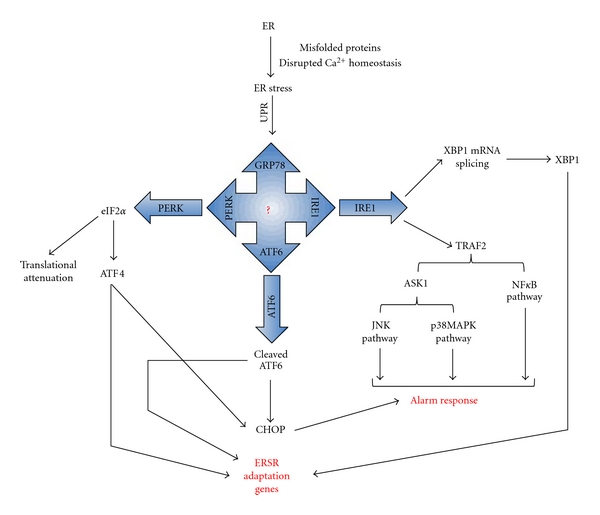
*Signal transduction events associated with ER stress*. The accumulation of misfolded proteins and disruption of Ca^2+^ homeostasis in the ER disrupt ER function leading to ER stress. The unfolded protein response (UPR) is initiated as a response to this stress, where GRP78, PERK, IRE1, and ATF6 play a central role. However, there might be more unknown mediators of ER stress. The cell initially tries to resolve the ER stress and restore normal cell function by halting protein synthesis and activation of several ER Stress Response (ERSR) adaptation genes, which include chaperones and proteins of the ER-associated degradation (ERAD) system. However, prolonged ER stress leads to the activation of the “alarm response”, leading to cell damage, dysfunction, and finally apoptosis.

The UPR engages to alleviate ER stress by (1) transcriptional induction of UPR genes (e.g.: chaperones to enhance folding capacity), (2) translational attenuation of global protein synthesis (to reduce the ER workload and thus decreases further production of misfolded proteins), and finally (3) ER-associated degradation (ERAD, to remove and clear unfolded proteins from the ER lumen) [65]. This tripartite mode of response ensures that, in the event that the stress responses are insufficient to restore the ER environment (ER stress remains unresolved with an environment suboptimal for proper protein folding), the cell is destined for programmed cell death/apoptosis [66, 72–74]. At the molecular level the three main proximal transducers of ER stress are: (1) protein-kinase-like ER Kinase (PERK), (2) inositol requiring protein-1 (IRE1), and (3) activating transcription factor-6 (ATF6) [65] (Figure 2).

**Figure 3:**
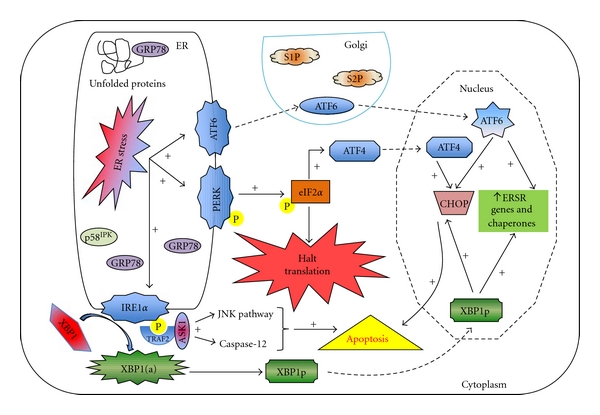
*Signaling by PERK, IRE1, and ATF6*. In an unstressed condition, GRP78, the primary sensor of ER stress binds to transmembrane ER proteins, PERK, IRE1, and ATF6, preventing their activation. The accumulation of unfolded proteins leads to dissociation of GRP78 from PERK, IRE1, and ATF6. GRP78, however, binds to the unfolded protein. GRP78 dissociation leads to PERK and IRE1 oligomerization and trans-autophosphorylation of their cytosolic domains. The active phosphorylated PERK (p-PERK, active) in turn phosphorylates eIF2*α* (p-eIF2*α*, inactive), leading to attenuation of global protein synthesis. Under these conditions, selected mRNAs, such as ATF4, are translated, which induces the expression of genes involved in restoring ER homeostasis. ATF4 can activate GADD34, which recruits a phosphatase to dephosphorylate p-PERK and reverse translational attenuation. ER-resident p58^IPK^s is an inhibitor of PERK. Phosphorylation of IRE1 (p-IRE1) activates its endoribonucleolytic activity, which then excises an intron of the XBP1 mRNA to generate a mature XBP1 mRNA, which then encodes for the active protein, XBP1. The XBP1 protein then translocates into the nucleus and supports the ER stress response. Interaction of p-IRE1 with TRAF2 can elicit the activation of the JNK pathway and caspases leading to apoptosis. GRP78 release from the dimeric ATF6 leads to the translocation of its monomeric form to the Golgi, where it undergoes proteolytic processing by the proteases (S1P and S2P). The cleaved ATF6 then translocates to the nucleus where it activates the ERSR genes and chaperones. Alternatively, CHOP/GADD153 can be activated by ATF4, ATF6, or XBP1, which promotes apoptosis by decreasing the levels of antiapoptotic Bcl-2 in the cell. The dotted arrows denote translocation. XBP1 (a) denotes the mature XBP1 mRNA, while XBP1p denotes the protein product of XBP1 (a). The “+” sign signifies activation.

PERK, an ER transmembrane, dimerizes and undergoes trans-autophosphorylation upon ER stress. The active phosphorylated from of PERK phosphorylates the *α*-subunit of eukaryotic translation initiation factor 2-alpha (eIF2*α*), leading to reduced recognition of AUG initiation codons, translational attenuation, and thus reduction of unfolded proteins in the ER [53, 60, 63]. To ensure that the translational attenuation is reversed once the ER stress is relieved, the translation of mRNA encoding genes such as activating transcription factor-4 (ATF4), which have a lower requirement of eIF2, is enhanced [61]. ATF4 then induces growth arrest and DNA damage-inducible protein-34 (GADD34), which in turn recruits protein phosphatase-1 (PP1) to dephosphorylate the PERK-phosphorylated eIF2*α* thus reversing translational attenuation [61, 75] (Figure 3).

IRE1, a ubiquitously expressed ER transmembrane glycoprotein, is unique in the fact that it contains both kinase and endoribonuclease activities in the cytoplasmic domain [76]. Oligomerization and trans-autophosphorylation of IRE1 in response to ER stress activates its endoribonucleolytic activity, which then specifically excises an intron of the X-box binding protein-1 (XBP1) mRNA to generate a mature XBP1 mRNA [77]. This XBP1 mRNA with a new open reading frame encodes for a 376aa protein and a C-terminal transcriptional activation domain and translocates to the nucleus which then induce numerous ER stress response genes [61, 76–78] (Figure 3).

ATF6 (670aa), though an ER transmembrane like PERK and IRE1, exists as a dimer (*α* and *β*) in the absence of ER stress [61–63, 65]. ER stress facilitates the translocation of the monomeric forms of ATF6 (~90 kDa) to the Golgi lumen where they are cleaved by the S1P and S2P proteases, yielding N-terminal cytosolic (~400aa, 50 kDa) fragments, which translocate to the nucleus and drive the activation of many ER stress response genes [61–63, 65] (Figure 3).

Although, PERK, IRE1, and ATF6 efficiently relay information to the nucleus regarding the accumulation of misfolded proteins in ER stress, the master regulator and sensor or ER stress seems to be the glucose regulated protein-78 (GRP78) [65]. GRP78 serves as a negative regulator of PERK, IRE1, and ATF6 by interacting with the luminal domains of these proteins in the absence of stress. The accumulation of unfolded proteins in the ER leads to the release of GRP78 (which binds to the unfolded proteins) from the transducers followed by subsequent activation of the UPR [61, 66]. While GRP78 dissociation form PERK and IRE1 permits their dimerization and activation, the release of GRP78 from the dimeric ATF6 permits its translocation to the golgi lumen where it is subjected to intramembrane proteolysis as mentioned earlier [79, 80] (Figure 3). 

In conditions where the stress causing the UPR cannot be resolved, leading to the continuous accumulation of unfolded proteins in the ER, the aim of the UPR switches gears from a prosurvival mode to that of a proapoptotic mode [65]. Although well-known pathways such as the NF*κ*B signaling, the JNK signaling pathway, and the p38MAPK pathway are known to play important roles in IRE1-mediated initiation of apoptosis, the contribution of each of these pathways or crosstalk between them requires further experimental clarification [59]. The proapoptotic CHOP/GADD153 is upregulated downstream of ATF4, which in turn downregulates the antiapoptotic Bcl-2, favoring mitochondrial cytochrome c release, caspase activation, and eventually apoptosis [81].

## 6. ER Stress and Endothelial Dysfunction in Diabetes—Possible Links

ER stress and the UPR play important roles in the pancreatic islet cell survival and function [82]. Accumulation of misfolded mutant insulin (in type I diabetes) in the ER of pancreatic *β*-cells causes chronic ER stress [83]. PERK overactivation in such cases and lack of p58^IPK^ (a PERK inhibitor), which is necessary in derepression of translation attenuation caused by PERK, are known to cause pancreatic *β*-cell apoptosis, leading to type I diabetes [84, 85]. Additionally, defective islet proliferation and increased ER stress-induced apoptosis were observed in PERK-deficient mice which are known to develop severe hyperglycemia soon after birth [86]. While mice on a high fat diet with heterozygous-targeted mutation of the PERK substrate, eIF2*α*, exhibit glucose intolerance, mice with homozygous mutation of the eIF2*α* phosphorylation site (Ser51Ala) die prenatally with diabetes and pancreatic *β*-cell deficiency [70, 87]. Obesity, one of the leading cause of type II diabetes, evokes ER stress in the peripheral tissues by activation of IRE1-induced JNK-dependent serine phosphorylation of IRS1, leading to insulin resistance [88]. While IRE1*α*−/− and XBP−/− knockout mice are not viable, XBP+/− mice are known to exhibit insulin resistance and type II diabetes [88–90].

The above-mentioned evidence, though suggestive of the possible role of ER stress in the development of diabetes, does not shed light on the role of ER stress and UPR in the vascular complications that follow hyperglycemia. The ER, exquisitely sensitive to glucose availability, depends on the blood glucose levels for the energy supply required for the protein folding process [91]. Endothelial cells, always exposed to elevations and reductions of blood nutrients, are very dynamic, metabolically active cells, with a high volume of protein synthesis, which predispose them to ER stress [92]. Endothelial cells in particular cannot tolerate the continued high glucose exposure, and thus ER stress is initiated in a diabetic milieu [93]. However, it is yet unclear whether endothelial dysfunction in response to high glucose levels occurs secondary to increased oxidative stress and a concomitant increase in ER stress or vice versa.

### 6.1. High Glucose-Induced Oxidative Stress and ER Stress—Double Edged Sword—Which Side Is Sharper?

Several studies suggest that hyperglycemia-induced oxidative stress contributes to significant endothelial dysfunction and vascular complications in diabetes (briefly reviewed under the subheadings Etiology of Endothelial Dysfunction in Diabetes and Molecular Basis of Endothelial Dysfunction in Diabetes—Current Understanding, in this paper). Although there are some studies suggesting that glucose-induced ER stress is independent of oxidative stress in endothelial cells [94], a large body of evidence suggest that oxidative stress induces ER stress and vice versa [91, 95]. Since the ER maintains an oxidative environment which favours protein folding and maturation, increase in the protein synthesis and protein folding load in response to hyperglycemia can lead to the accumulation of ROS [96]. In order to limit the accumulation of ROS, the PERK pathway is known to activate an antioxidant program (including the GSH) though ATF4 and NRF2 activation [96]. Hyperglycemia-mediated changes in the PERK pathway may thus contribute to ROS accumulation due to impaired antioxidant response. Recently, paraoxonase-2 (PON2), an ER-resident enzyme, is known to reduce ROS generation in the ER, thus moderating ROS-activated ER stress and reducing apoptosis of endothelial cells [97, 98].

As mentioned earlier, UPR first aims to restore cellular homeostasis by controlling the accumulation of misfolded proteins in the ER. In particular, GRP78, the ER stress-induced chaperone, is sensitive to glucose concentration and shown to be induced in endothelial cells [99]. ER stress inducers such as thapsigargin and tunicamycin are known to induce GRP78 expression in endothelial cells [92]. GRP78 induction may also protect human endothelial cells from oxidative stress-induced cellular damage [100].

NADPH oxidase, the prime source of ROS in endothelial cells is activated in presence of high glucose with a concomitant decrease in the generation of NO [26, 42–44, 101, 102]. Nox1/Nox2 activation (produce superoxide anions) upon high glucose exposure leads to the formation of detrimental levels of ROS which can salvage NO to form ONOO^−^ and cause eNOS uncoupling [103]. On the other hand, Nox4 (protective and produces H_2_O_2_ constitutively) activation leads to controlled ROS production which is known to play a significant role on endothelial cell signaling and vasodilatation [103]. NADPH oxidase (Nox1/Nox2) activation might link ER stress and oxidative stress to the high glucose-induced apoptosis of endothelial cells [104]. Nox2 activation and oxidative stress further amplify CHOP/GADD153 induction, which in turn promotes apoptosis. Reports suggest that CHOP induction and apoptosis as a response to ER stress is reduced in Nox2-deficient mice thereby preventing renal dysfunction [104]. This might be true for high glucose-exposed endothelial cells where Nox2 activation has shown to induce apoptosis [105] which might be possibly through the activation of CHOP-mediated ER stress response.

High glucose-induced ROS generation disrupt Ca^2+^ homeostasis leading to leakage of Ca^2+^ from the ER lumen [95, 106]. As mentioned earlier, intra-ER Ca^2+^ levels are critical, since many ER chaperones depend on Ca^2+^ for their activity [60]. A decrease in intra-ER Ca^2+^ can thus impair the UPR leading to a situation where the ER stress is not resolved when the cell must finally decide to undergo apoptosis [105]. In endothelial cells exposed to high glucose, disrupted Ca^2+^ homeostasis, due to ROS generation, can thus evoke an apoptotic response which may be induced by ER stress response mediators [107]. Additionally, increase in the cytosolic Ca^2+^ concentration can also stimulate mitochondrial ROS generation through various mechanisms [95]. Increased mitochondrial Ca^2+^ loading can generate ROS as byproduct of the electron transport chain and can mediate cytochrome c release and apoptosis [108]. Ca^2+^ may also stimulate the Krebs cycle in endothelial cells thereby increasing O_2_ consumption and ROS generation [109]. Ca^2+^- induced stimulation of eNOS can yield large amounts of NO, which can react with the superoxide anion to form ONOO^−^, thereby oxidizing BH_4_ and promoting eNOS uncoupling [106].

### 6.2. Hyperglycemia-Induced Endothelial Cell Apoptosis—Role of ER Stress

ER stress response transducers initiate apoptosis once resolution of the stress has not been achieved. However, the point at which the “apoptotic switch” is activated in response to ER stress has not yet been elucidated. ER responses to unfolded proteins (adaptation, alarm, and apoptosis) are primarily mediated through IRE1 [59]. IRE1 activation leads to the activation of XBP1 (adaptation), tumor necrosis factor receptor-associated factor 2 (TRAF2) TRAF2 (alarm through NF*κ*B), and JNK and p38MAPK pathways (apoptosis through ASK1 and caspase-12) [59, 73]. High glucose exposure of endothelial cells and high glucose-mediated oxidative stress in endothelial cells are known to activate ASK1 [110, 111]. ASK1 can also cause NO deficiency (a hallmark of endothelial cell dysfunction in diabetes) by regulating eNOS [112]. Oxidant stress-mediated ASK1 activation can cause downregulation of antiapoptotic Bcl-2, disruption of the mitochondrial membrane potential, and activation of a caspase cascade [113]. As mentioned earlier CHOP/GADD153 activates apoptosis by downregulating Bcl-2 [81]. Bcl-2 is known to be reduced in high glucose-exposed endothelial cells which may lead to the increase in intracellular AGE (characteristic of endothelial cell dysfunction in diabetes levels) [114, 115]. Linking the modulation of ASK1 and Bcl-2 levels in hyperglycemia, ER stress response might play a critical role in endothelial cell dysfunction in diabetes.

It has been shown that high glucose induces apoptosis of human endothelial cells through sequential activation of JNK and caspase-3 [116]. Studies suggest that caspase-12, an ER stress response mediator of apoptosis, can activate caspase-3 [73]. Blocking JNK and caspase-3 activity in high glucose-exposed human endothelial cells in culture prevented high glucose-induced apoptosis [116]. Additionally, vitamin C treatment (antioxidant) reduced JNK levels in these high glucose-exposed cells [116]. Given the role of ER stress response in JNK caspase-12 activation, the high glucose-induced ROS generation-mediated increase in endothelial cell apoptosis due to the activation of JNK and caspase-3 activity and its reversal with blockade of JNK/caspases-3 or antioxidant treatment indicate that ER stress might play a significant role in endothelial cell dysfunction in diabetes.

Tumor necrosis factor-*α* (TNF*α*), another known activator of the JNK pathway and inducer of apoptosis of endothelial cells, is known to be activated by high glucose-induced oxidative stress [117–120]. TNF*α* can in turn generate ROS and reduce activation of eNOS [121]. TNF*α* activation and its binding to its receptor TNFR1 recruit TRAF2 leading to downstream activation of NF*κ*B, JNK and caspases which in turn decides cell death and survival decisions [120]. Increase in the levels of TRAF2 has been shown in high glucose-exposed endothelial cells [122]. TRAF2, known to be essential for ER stress induced IRE1 mediated UPR, can be an important link for the role of ER stress in endothelial cell dysfunction in diabetes.

p58^IPK^ inhibits PERK, thereby inhibiting eIF2*α* phosphorylation and returning ER homeostasis in stressed cells [84]. p58^IPK^ transfection may have significantly reduced apoptosis of retinal endothelial cells by decreasing both mRNA and protein levels of CHOP and TNF*α* in the retina of diabetic rats, thereby reducing retinal blood vessel leakage [123, 124]. As in the case of pancreatic *β*-cells [85], prolonged exposure of endothelial cells to high glucose levels might decrease p58^IPK^ leading to ER stress-mediated endothelial cell dysfunction in diabetes.

### 6.3. Endothelial Cell Response to Insulin in Diabetes—Role of ER Stress

Cellular insulin resistance may be selective in terms of its nature and extent with respect to certain cell systems and may vary in terms of the metabolic, mitogenic, pro-survival, and vascular actions of insulin [16]. Protective effects of insulin include its ability to protect against apoptosis and to stimulate the production of NO [125, 126]. The role of insulin resistance at the level of the endothelial cell in vascular diseases is rather unclear.

The increase in the protein-folding demand and the signaling involving calcium and ROS induce the UPR, leading to the transcription of genes whose products mount an inflammatory response. An excess of glucose can further boost the UPR and inflammation, contributing to insulin resistance and apoptosis. The ER stress response-conferred insulin resistance in endothelial cells could also further promote inflammatory stress signaling and contribute to the metabolic deterioration that is associated with type II diabetes and vascular diseases. JNK and I*κ*B kinase (IKK) are activated (through an IRE-1-alpha dependent fashion), in addition to proinflammatory genes, in response to ER stress in these cells as well [82, 127]. Activated JNK phosphorylates serine residues on IRS-1, thereby inhibiting it, and the overall insulin-signaling pathway. This in turn leads to insulin resistance through defective downstream signaling (causing less AKT activation and lower NO production) [82, 128].

## 7. Potential Therapeutic Strategies

Studies have brought to light the possible link between hyperglycemia-induced ER stress and endothelial cell dysfunction. Given the relationship between oxidative stress and ER stress, the probable first line of therapy would be the use of biologically known and pharmacologically available antioxidants. Thioredoxin-1 (Trx1), an extensively studied antioxidant, growth regulator, and antiapoptotic protein, is known to interact with and inhibit ASK1 activity [27], one of the prime mediators of ER stress-related apoptotic response. Recently, it was reported that hyperglycemia-induced oxidative stress was primarily due to the induction of Trx1 inhibitory protein (TXNIP), an endogenous inhibitor of Trx1 activity, leading to the inhibition of the antioxidant function of Trx1 [129]. TXNIP binding to Trx1 further reduces the ability of Trx1 to bind efficiently with its other protein partners, such as ASK-1 thereby reducing the antiapoptotic property of Trx1 [129]. Studies are warranted to investigate the role of Trx1 and TXNIP in the induction of ER stress response to hyperglycemia in endothelial cells.

Preserving or restoring ER function might be therapeutic. Certain small molecules (chemical chaperones) such as 4-phenylbutyric acid and taurine-conjugated ursodeoxycholic acid were found to significantly reduce phosphorylation of PERK and IRE1*α* to improve glucose tolerance and insulin sensitivity [130]. A tetrameric form of resveratrol (vaticanol B) exhibited the capacity to inhibit UPR and inflammatory response by reducing protein-folding load and maintaining membrane integrity thereby preventing ER stress-induced apoptosis [131]. Salvianolic acid B was recently shown to protect endothelial cells from oxidative stress damage through the induction of GRP78 [100]. Salubrinal-induced dephosphorylation of eIF2*α* has shown to protect cells from ER stress-induced apoptosis [132].

ERAD involves the Ubiquitin-26S proteasome degradation pathway in the tagging and degradation of terminally unfolded proteins. Efficiency of the ubiquitin-proteasome system pathway in eliminating the unfolded proteins probably decides the fate of the cell [133]. A compromised ERAD/ubiquitin-proteasome system would mean that even though there has been a check on the synthesis of unfolded proteins through ER stress response, the cell is unable to get rid of accumulated misfolded proteins. This would mean that since the ER stress cannot be resolved the cell has to undergo apoptosis. Hyperglycemia is known to impair proteasome function [134]. The loss of function of the ubiquitin-proteasome system and continuing accumulation of unfolded proteins in the ER might be the “apoptotic switch” which when turned on demands the cell to switch gears from prosurvival mode to proapoptotic mode. Hence impaired ERAD/ubiquitin-proteasome system would play a significant role in hyperglycemia-induced ER stress and endothelial cell dysfunction. Therapeutic strategies to improve the efficacy of the ubiquitin-proteasome system thus could possibly confer protection from diabetes-associated endothelial cell dysfunction and oxidative stress.

## 8. Conclusion

In conclusion, the current paper suggests the role of diabetes-mediated ER stress in the endothelial cell dysfunction and accompanying cardiovascular diseases in diabetes. These possible links between hyperglycemia-induced ER stress and oxidative stress might offer the premises for conducting additional experiments to establish a unified molecular mechanism and thereby identify a potential therapeutic target in reducing vascular complications in diabetes. Elucidation of this molecular mechanism could also extend this to other pathological conditions where ER stress and oxidative stress might play a causal role. Thus a direct response to the current paper would be to test potential therapeutic agents (antioxidants and/or chemical chaperones) that might aid in long-term restoration of the endogenous capacity of endothelial cells to initiate and ER stress response in response to high glucose-induced protein misfolding, restore ER homeostasis, and escape apoptosis.

More studies are required to establish a time-dependent variation in the expression of ER stress proteins in a diabetic milieu. It is possible that in early stages of diabetes the endothelial cell may compensate for the ER stress that is associated with hyperglycemia while in later stages of advanced diabetes these compensatory mechanisms fail resulting in endothelial cell dysfunction and associated complications. Moreover, studies are required with adequate glycemic controls to examine whether the extent or duration of hyperglycemia could possibly regulate the expression of ER stress transducers.

## Abbreviations

AGEs: Advanced glycation end products
ANG-II: Angiotensin II
ASK1: Apoptosis signal regulating kinase-1
ATF4: Activating transcription factor-4
ATF6: Activating transcription factor-6
BH_4_: Tetrahydrobiopterin
CHOP: CCAAT/-enhancer-binding protein homologous protein/GADD153
DM: Diabetes mellitus
eIF2*α*: Eukaryotic translation initiation factor 2-alpha
eNOS: Endothelial nitric oxide synthase
ER: Endoplasmic reticulum
ERAD: ER-associated degradation
ERSR: ER stress response
ET-1: Endothelin-1
GADD153: Growth arrest and DNA Damage-inducible protein-153/CHOP
GADD34: Growth arrest and DNA Damage-inducible protein-34
GAPDH: Glyceraldehyde phosphate dehydrogenase
GSH: Glutathione
HAECs: Human aortic endothelial cells
ICAM-1: Intercellular adhesion molecule-1
IKK: I kappa B kinase
iNOS: Inducible nitric oxide synthase
IRE1: Inositol-requiring protein-1
JNK: c-Jun N terminal kinase
MCP-1: Monocyte chemoattractant protein-1
NF*κ*B: Nuclear factor kappa B
NO: Nitric oxide
O_2_^∙−^: Superoxide anion
ONOO^−^: Peroxynitrite
PAI-1: Plasminogen activity inhibitor-1
PARP: Poly(ADP) ribose polymerase
PERK: Protein-kinase-like ER kinase
PKC: Protein kinase C
PP1: Protein phosphatase-1
ROS: Reactive oxygen species
TNFR1: TNF*α* receptor-1
TNF*α*: Tumor necrosis factor alpha
TRAF2: TNF receptor-associated factor-2
Trx1: Thioredoxin-1
TXNIP: Trx interacting protein
UPR: Unfolded protein response
VCAM-1: Vascular cell adhesion molecule-1
VSMCs: Vascular smooth muscle cells
vWF: von-Willebrand Factor
WHO: World Health Organization.
